# Single cell ICP-MS for the assessment of potential nephroprotectors against cisplatin

**DOI:** 10.1007/s00604-025-07383-8

**Published:** 2025-07-23

**Authors:** Alejandro Iglesias-Jiménez, Gema Artiaga, Estefanía Moreno-Gordaliza, Pilar Bermejo-Barrera, Antonio Moreda-Piñeiro, M. Milagros Gómez-Gómez

**Affiliations:** 1 Department of Analytical Chemistry, Faculty of Chemistry, Universidad Complutense de Madrid, Avenida Complutense S/N, 28040 Madrid, Spain; 2 Group of Trace Elements, Spectroscopy and Speciation (GETEE), Institute of Materials iMATUS, Department of Analytical Chemistry, Nutrition and Bromatology. Faculty of Chemistry, University of Santiago de Compostela, Avenida das Ciencias, S/N, 15782 Santiago de Compostela, Spain

**Keywords:** Cisplatin, Kidney protection, Selenium nanoparticles, Selenomethionine, Methionine, Single cell ICP-MS

## Abstract

The use of cisplatin chemotherapy is often limited by the occurrence of various side effects, with renal toxicity being one of the most serious. In the present work, a single cell ICP-MS (scICP-MS) methodology was optimised to evaluate the cellular uptake of cisplatin in the presence of three potential nephroprotectors such as chitosan-stabilised selenium nanoparticles (Ch-SeNPs), selenomethionine (SeMet) and methionine (Met). Human telomerase reverse transcriptase-immortalised renal proximal tubular epithelial cells (RPTEC/TERT1) and human cervical cancer cells (HeLa) were employed with this aim. In both cell lines, a decrease in the intracellular Pt levels when using SeMet and Met as coadjuvants was revealed, involving less toxicity in renal cells but no reduction in the anticancer effect after measurement of cell viability by MTT assays. In contrast, Ch-SeNPs had no effect on the internalisation of the Pt-drug but enhanced its antitumour efficacy with no additional damage to kidney cells. This would allow decreasing cisplatin doses which would in turn reduce nephrotoxicity risk. Se determination by scICP-MS was also done to study the cell uptake of the selenocompounds, in addition to transmission electron microscopy (TEM) analysis of Ch-SeNPs internalisation. The effects of both SeMet and Ch-SeNPs were confirmed despite the Pt-drug was shown to induce a decrease in cell uptake. Results were compared by two different scICP-MS settings (a conventional introduction system and a special configuration for intact cells), as well as with the classical digestion-based bulk analysis. Our results demonstrate the potential of scICP-MS for metallomic cellular studies to improve cisplatin-based therapies.

**Graphical Abstract:**

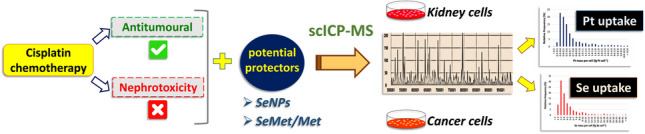

**Supplementary Information:**

The online version contains supplementary material available at 10.1007/s00604-025-07383-8.

## Introduction

Nephrotoxicity is often the most important limiting side effect of cisplatin-based anticancer therapies, with nearly one in three patients failing to complete treatment after developing acute kidney injury [1]. Although many compounds have been evaluated to replace cisplatin, its administration is still required in many cases where it provides the best antitumour efficacy [2, 3]. Current research is therefore focused not only on the development of valid substitutes, but also on therapies that combine the Pt-drug with a potential renoprotector that does not interfere with its anticancer activity [4]. Selenium has been tested for this purpose, as its antioxidant nature could reduce the oxidative damage suffered by kidney cells exposed to the Pt-drug [5], with selenium nanoparticles (SeNPs) and the amino acid selenomethionine (SeMet) being two of the Se-based compounds with better prospects. Diverse studies have shown that SeNPs could be useful for antimicrobial, fungicidal, antiviral, anti-inflammatory, or anticancer applications, but also for minimising the nephrotoxicity caused by various agents such as cisplatin in both cell and animal models [6, 7]. On the other hand, the protective potential of SeMet has been demonstrated in rats and mice, since its co-administration with cisplatin led to a reduction in renal damage, together with a reduced accumulation of the Pt-drug [8, 9]. Methionine (Met), the sulphur analogue of SeMet, has also been shown to reduce the toxicity and uptake of cisplatin in kidney cells, confirming the influence of the interaction between Pt and both Se and S on the biological effect of this drug [10–13].

The search for new strategies to improve current therapies requires the understanding of the behaviour of the antitumoural compounds and the potential protectors at the cellular level. In this sense, metallomic analyses based on mass spectrometry have proven to be a powerful resource, allowing quantification, speciation and distribution studies of metallic or metalloid-based compounds in complex biological samples [14]. In the last years, single cell ICP-MS (scICP-MS) has emerged as a potential alternative for trace element determination in cells, using the same principles as single particle ICP-MS (spICP-MS), which has recently revolutionised nanometrology due to its ability to measure intact nanocolloids by time-resolved analysis (TRA) [15, 16]. The use of more efficient sample introduction systems with better preservation of cell integrity, lower sample volumes (a few microlitres per minute) and shorter dwell times (within the microsecond range) enables scICP-MS to measure elemental content in individual cells, behaving as an elemental flow cytometer. In this way, the characteristic intensity pulses corresponding to the intracellular mass of the analyte in individual cells can be distinguished from the continuous signal produced by the background noise and a possible extracellular contribution. This makes scICP-MS useful for speciation purposes, similarly to the differentiation between nanoparticles and dissolved elements achieved with spICP-MS. Obtaining mass distributions for the internalised analytes provides a better knowledge of the samples from a statistical point of view, considering the large differences that can be found in the behaviour of cells from a single population. This is an important advantage over traditional bulk ICP-MS analysis of digested samples, which provides only an average content of a cell population [17]. The analytical potential of scICP-MS has been demonstrated in various studies on cellular internalisation, accumulation and release of a wide range of compounds, from molecules to nanoparticles, in different cell lines as well as bacteria, yeast or unicellular algae [18–27].

A scICP-MS method has been optimised for the determination of the cellular uptake and accumulation of cisplatin in co-treatments with chitosan-functionalised SeNPs (Ch-SeNPs), SeMet and its sulphur analogue Met. In order to get a more comprehensive understanding of the treatment effects, an integrative approach was employed, combining scICP-MS analyses of the intracellular content of Pt and Se, and biological assays such as cell viability assays (MTT) and transmission electron microscopy (TEM-EDS) to assess nanoparticle internalisation and cell morphology. Human telomerase transcriptase-immortalised renal proximal tubular epithelial cells (RPTEC/TERT1) and human cervical cancer cells (HeLa) exposed to the agents were used in an attempt to investigate their antitumour action and the potential nephroprotection, scICP-MS.

Here, a scICP-MS method has been optimised for the determination of the cellular uptake and accumulation of cisplatin in co-treatments with chitosan-functionalised SeNPs (Ch-SeNPs), SeMet and its sulphur analogue Met as potential nephroprotectors. For this aim, human telomerase transcriptase-immortalised renal proximal tubular epithelial cells (RPTEC/TERT1) and human cervical cancer cells (HeLa) were employed, in an attempt to investigate the two sides of the above treatments: antitumour activity and kidney protection. Electron microscopy (TEM-EDS) was employed to characterise the synthetised SeNPs and also to confirm their internalisation by cells. The study was complemented by the measurement of cell viability by MTT assays to assess the applicability of the different compounds for nephroprotective strategies. Finally, the results corresponding to scICP-MS analyses of the intracellular content of Pt and Se were compared to those obtained with bulk ICP-MS measurements after acid digestion of cell pellets.

## Materials and methods

### Preparation of selenium nanoparticles (SeNPs)

#### Uncovered SeNPs

First, 312.5 µL of an aqueous solution of 0.1 M sodium selenite (Sigma-Aldrich, St. Louis, MO, USA) was mixed with 5 mL of ultrapure water using gentle and continuous magnetic stirring, before slowly adding 143 µL of 0.35 M ascorbic acid (Sigma-Aldrich). The mixture was then diluted with water to a final volume of 25 mL. Stirring was kept overnight until the solution turned bright orange, indicating the reduction of dissolved Se(IV) to colloidal Se(0). At the end of this procedure, SeNPs were purified by dialysis in cellulose membranes with a MWCO of 14 kDa (Sigma-Aldrich) against 1.5 L of ultrapure water for 3 h, changing the dialysis medium to fresh water after every hour. The final solution was stored at 4 °C.

#### Chitosan-functionalised SeNPs (Ch-SeNPs)

This synthesis was based on the method published by Bai et al. [28], where full characterisation of the Ch-SeNPs was performed. Briefly, aqueous solutions of ascorbic acid (4.93 mL, 0.35 M) and acetic acid (5 mL, 0.96 M) (Panreac AppliChem, Barcelona, Spain) were mixed under magnetic stirring, and then 10 mL of 0.5% (m/v) chitosan (Sigma-Aldrich) was added. Then, 1.35 mL of 0.1 M sodium selenite was added very slowly. Ultrapure water was then added to the mixture to a final volume of 45 mL under stirring for a further 5 min. Unlike the previous synthesis, the colour of the resulting solution changed very quickly to a deep red. The resulting Ch-SeNPs were dialysed and stored as described above for uncovered SeNPs.

### Characterisation of SeNPs

#### Microscopy analysis of nanoparticles

Transmission Electron Microscopy-Energy Dispersive X-ray Spectroscopy (TEM-EDS) was used to study the morphology, degree of aggregation and elemental composition of the particles prepared. Drops of each suspension were placed on copper grids with the side covered with carbon. Analyses were carried out using a JEM 2100 microscope (JEOL, Tokyo, Japan) at 200 kV. The diameters estimated for > 100 particles were registered to create size distribution histograms for both uncapped SeNPs and Ch-SeNPs. Size measurements were done with the software ImageJ (https://imagej.nih.gov/ij/).

#### Determination of Se concentration in SeNPs by ICP-MS

The Se concentration in SeNPs was determined after acid digestion of each nanoparticle dispersion. First, 250 µL of the suspension was mixed with 750 µL of HNO_3_ (65%, Scharlab, Barcelona, Spain) and 250 µL of H_2_O_2_ (33%, Panreac AppliChem) in a PTFE digestion vessel (CEM Corporation, Matthews, NC, USA). Samples were then heated in a MARS microwave oven (CEM Corporation) using the following ramp temperature programme: 20 min increase from room temperature to 130 °C, followed by 15 min at 130 °C and final cooling to room temperature. After digestion, samples were diluted with ultrapure water until < 1% (v/v) HNO_3_ was achieved. This procedure was repeated in quintuplicate for each dispersion. Se quantification was performed by external calibration with aqueous standards ranging from 0.5 to 200 µg L^−1^, prepared from a certified Se solution of 1000 mg L^−1^ (TraceCERT, Merck, Darmstadt, Germany). Yttrium standard solution (1000 mg L^−1^, TraceCERT, Merck) was added to a final concentration of 1 µg L^−1^ as an internal standard. The analysis was performed on an Agilent 7700x ICP-MS (Agilent Technologies, Santa Clara, CA, USA), tuned daily with a tuning mixture containing Li, Co, Y, Ce and Tl (Agilent Technologies). The instrumental conditions are shown in Table S1.

### Cell incubations with cisplatin and the potential nephroprotectors

RPTEC/TERT1 cells (Evercyte, Vienna, Austria) were cultured with ProxUp medium (Evercyte), while DMEM (Dulbecco’s Modified Eagle’s Medium) medium from Gibco (Life Technologies, Thermo Fisher Scientific, Waltham, MA, USA) supplemented with 10% (v/v) fetal bovine serum (FBS) (Gibco) was used for HeLa. In both cases, cells were seeded in P100 plates (Corning Inc., Corning, NY, USA) and placed in a CO_2_ incubator (Thermo Fisher Scientific, Waltham, MA, USA) at 37 °C and 5% CO_2_ until they reached > 70% cell confluence. The cells were then exposed to different treatments for 24 and 48 h: cisplatin (30 µM), Ch-SeNPs (60 µM as Se), SeMet (60 µM), Met (60 µM), and cisplatin combined with each potential protector at the same concentrations as above. Cultures with uncapped SeNPs (60 µM as Se) and sodium selenite (Se(IV)) (60 µM) as well as control cultures (untreated cells) were also performed for comparative purposes, particularly during the evaluation of Se uptake by scICP-MS. Cisplatin, SeMet and Met were purchased from Sigma-Aldrich.

### Collection of pellets after cell incubations

Cell pellets were obtained from cultures in P100 plates for further analysis by ICP-MS. At the end of the incubations, the culture medium was discarded from the plates, and they were rinsed three times with phosphate-buffered saline (PBS) (Gibco). Trypsin/EDTA solution (Gibco) was then added to the plates, and they were kept in a CO_2_ incubator (37 °C, 5% CO_2_) for 5 min. The resulting detached cells were collected in fresh medium, and their concentration was measured using a *Countess II* automated cell counter (Invitrogen, Thermo Fisher Scientific, Waltham, MA, USA) after mixing 10 µL of cell suspension with the same volume of trypan blue solution (Sigma-Aldrich). The cells were then centrifuged at 230 g and 4 °C for 10 min to remove residual trypsin and resuspended in 1 mL of PBS. Finally, the supernatant was discarded by centrifugation (230 g, 4 °C, 5 min), and the cell pellets were stored in Eppendorf tubes (Eppendorf, Hamburg, Germany) at − 80 °C until analysis.

### Measurement of cell viability

3-[4,5-dimethylthiazol-2-yl]−2,5 diphenyl tetrazolium bromide (MTT) assays were used to quantify cell viability after the different treatments in P96 plates. First, cells were detached by trypsinization from a confluent P100 plate and counted as indicated above. Then, cells were seeded in a P96 plate (around 50,000 cells well^−1^) and incubated for 24 h, prior to the administration of cisplatin (30 µM) and the potential protectors (60 µM) (200 µL well^−1^), preparing five replicates for each case. After an exposure time of 24 or 48 h, MTT solution (2 mg mL^−1^) was added (50 µL well^−1^) and the plate was incubated for 4 h at 37 °C in the dark. Then, the supernatant was removed from the wells, and the resulting formazan crystals were dissolved by the addition of DMSO (100 µL well^−1^). MMT and DMSO were acquired from Sigma-Aldrich and Scharlab, respectively. The absorbance at 595 nm was measured in each well with a *Sunrise* microplate reader (Tecan, Männedorf, Zurich, Switzerland). Finally, cell viability was calculated from the ratio between the absorbance values of treated cells and controls. One-way analyses of variance (ANOVA) and two-tailed Student’s *t*-tests with a 95% confidence level were applied to compare cell viability results (*n* = 5), using the Analysis ToolPak of Microsoft Excel Professional Plus 2019 software.

### TEM-EDS analysis of cells exposed to NPs

TEM-EDS analyses were performed to confirm the internalisation of nanoparticles by cells. For this purpose, pellets of both RPTEC/TERT1 and HeLa were used, which were previously exposed to either unfunctionalised SeNPs or Ch-SeNPs (60 µM as Se in both cases) for 24 h in P100 plates. Briefly, cells were fixed with a solution containing p-formaldehyde and glutaraldehyde, washed with PBS, incubated as well in PBS at 4 °C overnight and stained with a solution of osmium tetroxide for 1 h in the absence of light. After being washed with ultrapure water, the samples were progressively dehydrated by successive incubations with acetone:water mixtures with increasing acetone content. The cells were then incubated with various Spurr resin:acetone solutions until 100% resin was reached. The final incubation in pure resin was maintained at 70 °C for 48 h. Finally, the cell resins were cut into thin sections using an ultramicrotome, stained with uranyl acetate and chromium citrate and placed on copper grids for TEM-EDS analysis using a JEM 1400 microscope (JEOL, Tokyo, Japan).

### Acid digestion of cell pellets and bulk analysis of intracellular Pt and Se by ICP-MS

After each cell pellet wash (1–4 times) with 1 mL PBS, cells were counted with a Countess II automated cell counter, resuspended in 250 µL of ultrapure water and transferred to PTFE digestion vessels before adding 750 µL of HNO_3_ (65%) and 250 µL of H_2_O_2_ (33%). Samples were then placed in a microwave oven and subjected to the same digestion programme as for SeNPs (section *Determination of Se concentration in SeNPs by ICP-MS*). Finally, the digested cells were diluted with ultrapure water up to 1% (v/v) HNO_3_. ICP-MS measurements, as described in Table S1, a, were performed to determine the content of Pt and Se in the samples. Calibration was performed with aqueous standards of Pt (0.05–20 µg L^−1^) and Se (0.5–200 µg L^−1^), using Ir (1 µg L^−1^) and Y (1 µg L^−1^) as internal standards, respectively. All the standards were prepared from certified solutions of each individual element of 1000 mg L^−1^ (TraceCERT, Merck). The mass of Pt or Se determined for each pellet was divided by the corresponding number of cells, and the value was expressed as the mean ± the standard deviation (sd) for three replicates.

### Single cell ICP-MS (scICP-MS) analysis of intracellular content of Pt and Se

For this study, cell pellets (> 2 × 10^6^ cells) were washed once or twice with 1 mL of PBS, then resuspended in 1 mL of ultrapure water and finally subsequent dilutions were also prepared in water just prior to the analysis (1:10, 1:20, 1:50, 1:100, 1:200 and 1:500). TRA-independent measurements of ^195^Pt and ^78^Se in the samples were performed using a NexION 2000 ICP-MS (Perkin Elmer, Waltham, MA, USA) specifically configured for single cell analysis: Asperon spray chamber, CytoNeb nebuliser, automated injection through syringe pumps and Syngistix Single Cell Application software module for data acquisition and processing. The ICP-MS was tuned daily prior to cell analysis using a NexION setup solution containing 10 µg L^−1^ of Be, Ce, Fe, In, Li, Mg, Pb and U (Perkin Elmer). Cell suspensions were transferred to 1 mL cylindrical cuvettes and then aspirated and resuspended by the autosampler, with 100 µL being the volume taken for analysis. The sample flow rate was set to 10 µL min^−1^, and 100 s was the total time for each measurement. Different dwell times were evaluated: 50, 100, 150 and 200 µs. A washing step was performed after each injection with an aqueous solution containing 1% (v/v) HNO_3_ and 2% (v/v) H_2_O_2_. The instrumental conditions used are given in Table S1, b. Intracellular contents of Pt and Se were quantified independently by external ionic calibrations, with solutions of 0.1, 0.2, 0.5, 1, 2, 5 and 10 µg L^−1^ prepared in ultrapure water by dilution of the corresponding certified 1000 mg L^−1^ standards of Pt and Se.

The calculations were done by the Syngistix Single Cell/Nano application through fast scanning data processing, a method designed for TRA measurements in the order of microseconds (µs). Briefly, the signal intensity of background is first determined after an iterative removal of all the data points which exceed the sum of the average intensity in the measurement plus *k* sd (standard deviation), where *k* is equal to or higher than 3. Then, a smoothing process is applied to the original dataset, and points corresponding to single cell peaks are identified when their intensity is higher than the threshold value indicated above. Once recognised, the peak area associated is calculated in all cases after subtracting the contribution of background signal for the time segments constituting each peak. To finish, the intracellular mass of element per cell is obtained using Eq. 1:

1 $${m}_{c}= \frac{\varepsilon \bullet {Q}_{sam} \bullet {A}_{peak}}{m}$$

where *m*_*c*_ is the mass of element of interest in a single cell, *ε* is the transport efficiency to reach the plasma, *Q*_*sam*_ is the sample uptake rate, *m* is the calibration slope and *A*_*peak*_ is the area under the curve of the single cell event after background correction.

The transport efficiency was determined using a solution containing 120 ng L^−1^ of certified 50 nm AuNPs (NanoComposix, San Diego, CA, USA) and was higher than 40% in all cases. The number of registered cell events, the mass of element per cell and the concentration of extracellular element were automatically provided by the software and verified by manual calculations, as well as the limits of detection (LOD), resulting in 6 ± 1 ag Pt cell^−1^ and 57 ± 12 ag Se cell^−1^. For comparison, analyses were also performed in TRA mode using a NexION 300X ICP-MS (Perkin Elmer) with no specific settings for the study of intact cells (Table S1, b). In this case, a conventional introduction system based on a cyclonic spray chamber and a concentric nebuliser was used with a flow rate of 100 µL min^−1^ through a peristaltic pump. Calibration with ionic standards and determination of transport efficiency (< 8%) were performed as previously described with the NexION 2000 model. The LOD in this case were 15 ± 2 ag Pt cell^−1^ and 128 ± 21 ag Se cell^−1^.

## Results and discussion

### TEM-EDS characterisation of SeNPs and their cellular internalisation

The results of the analysis of unfunctionalised SeNPs and Ch-SeNPs by TEM-EDS are shown in Figure S1. For both cases, Se-based particles were found to be spherical and showed a very low degree of aggregation (Fig. S1, a, b, e, f). The presence of a stabilising agent such as chitosan had a strong effect on the size of the particles, resulting in diameters around 42 nm for Ch-SeNPs (Fig. S1, h), in agreement with those reported by Bai et al. [28], whereas a broader size distribution centred around 120 nm was found for uncapped SeNPs (Fig. S1, d). This may be due to the formation of Se-N and Se-O bonds between HSe- and Se^2−^ anions in the particle surface and -NH_2_ and -CH_2_OH groups in the polysaccharide once the initial Se(IV) is reduced to colloidal Se(0). Such interaction would be favoured by the protonation of the amino groups in the acidic medium used for synthesis [29, 30]. These aspects may explain why the chitosan coating prevented higher aggregation and growth of the SeNPs, showing good stability up to 30 days after their synthesis, whereas unfunctionalised SeNPs started to collapse after 2 weeks. On the other hand, the stability of SeNPs did not seem to be affected when incubated for 24 and 48 h in the culture media (data not shown).

**Fig. 1:**
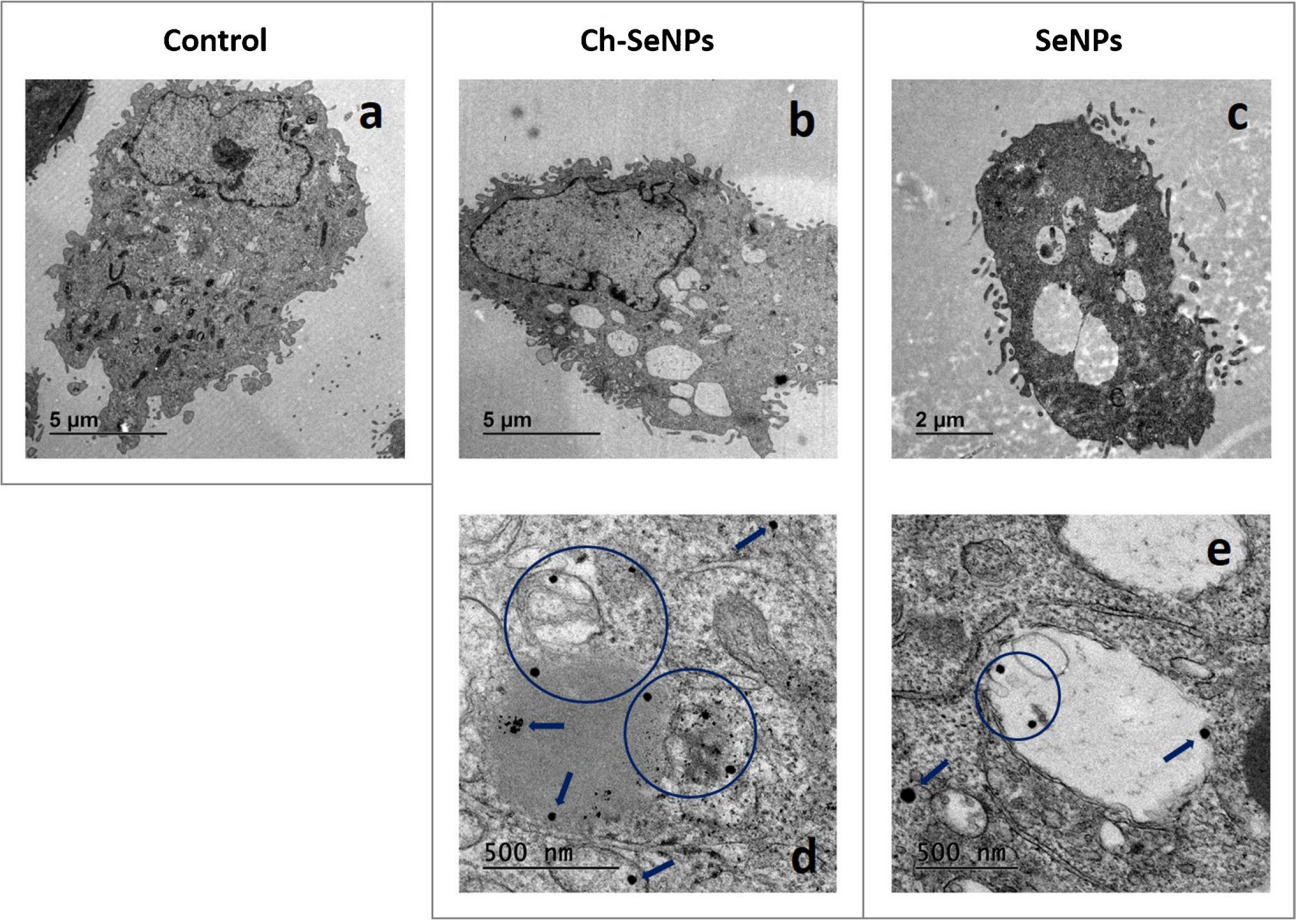
Microscopy images of RPTEC/TERT1 by TEM analysis: control cells (**a**), cells after 24 h exposure to Ch-SeNPs (**b**, **d**), and cells after 24 h exposure to uncovered SeNPs (**c**, **e**). Ch-SeNPs (**d**) and uncapped SeNPs (**e**) internalised by cells and located in different cell compartments such as the cytoplasm, vacuoles or mitochondria

The ultrastructure of cells cultured with Ch-SeNPs and uncapped SeNPs was subjected to TEM-EDS analysis to verify the cellular uptake of these compounds. As can be seen in the micrographs of RPTEC/TERT1 in Fig. 1, the number and dimensions of intracellular vacuoles increased (Fig. 1b–e) compared to non-treated cells (control cells) (Fig. 1a), with no further apparent changes in cell morphology. Internalisation of both types of SeNPs was confirmed as they were found in different intracellular locations, mainly in the cytoplasm and vacuoles (Fig. 1d, e). Ch-SeNPs were also found in lysosomes or mitochondria, and to a greater extent than uncoated SeNPs. Thus, as expected, the smaller size of Ch-SeNPs may facilitate not only their uptake by cells, but also their further incorporation into organelles. The presence of particles inside intracellular vacuoles, along with the increased formation of these organelles, seems to be consistent with an endocytic uptake rather than a diffusion process, as previously proposed for SeNPs with diameters similar to those used herein [31, 32]. Although a few SeNPs were also observed attached to the outer side of the plasma membrane (data not shown), these were almost negligible compared to the intracellular particles. This indicates efficient internalisation of the NPs without significant loss due to adsorption on the cell surface. Similar findings were done with the TEM images corresponding to HeLa (data not shown). For further experiments studying the renoprotective potential of SeNPs in the presence of cisplatin, only Ch-SeNPs were selected based on their higher stability over time and their favoured cell uptake.

### Effect of the potential nephroprotectors on cell viability

The concentration employed for cisplatin (30 µM) was selected from previous works of our group related to the study of nephroprotective strategies [33, 34]. This value resulted in a sufficiently high cell damage to be quantified by MTT assays, but still compatible with the detection of a potential effect on cell viability of the coadjuvants. Regarding Ch-SeNPs, SeMet and Met, preliminary MTT assays were performed to evaluate them at different concentrations in the absence of cisplatin (data not shown). No changes were observed for cultures with the two amino acids compared to control cells, whereas Ch-SeNPs at a concentration equal to or higher than 60 µM reduced HeLa viability. This did not occur for RPTEC/TERT1, but higher values were discarded to reduce the risk of cell cycle arrest associated with these particles [35]. Taking advantage of the specific cytotoxicity of Ch-SeNPs against tumour cells at 60 µM without damaging kidney cells, though our actual interest was their protective potential, we decided to use this concentration. Then, to compare the three coadjuvants, the same value (60 µM as Se) was applied in all cases for further experiments studying the renoprotective potential against cisplatin (30 µM).

**Fig. 2:**
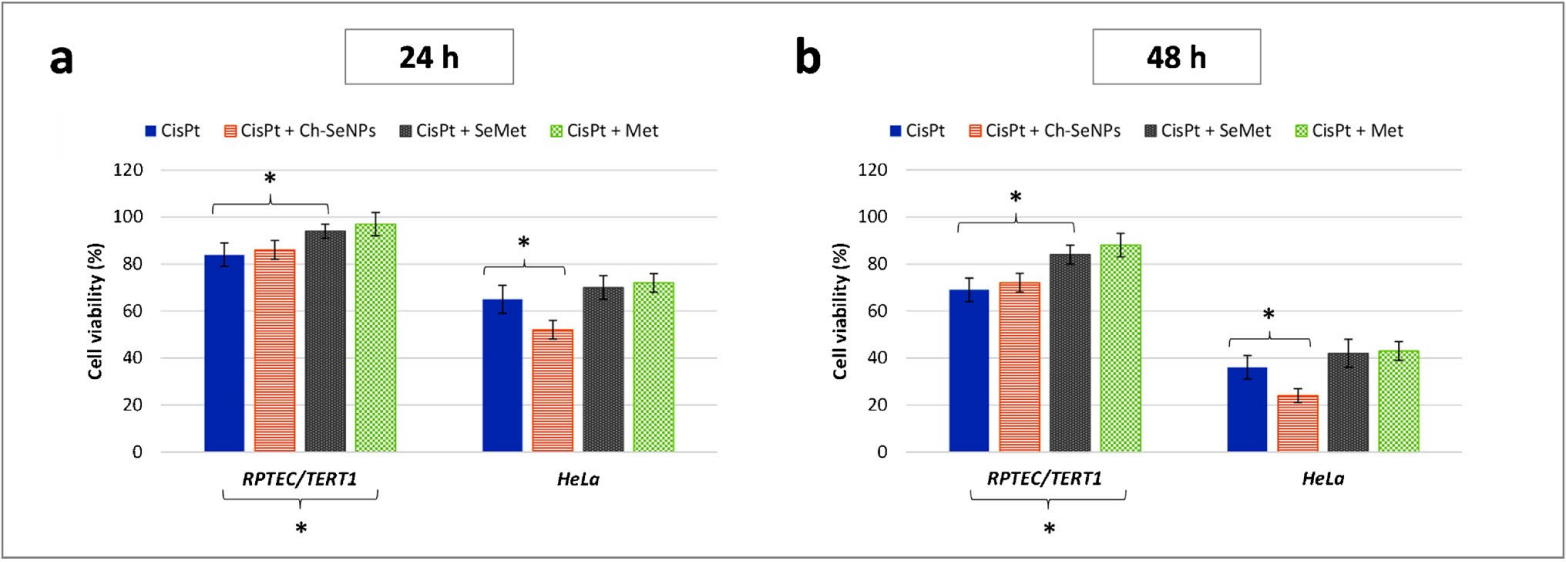
Cell viability results from MTT assays of 24 (**a**) and 48-h cultures (**b**) of RPTEC/TERT1 and HeLa after being exposed to cisplatin alone or combined with Ch-SeNPs, SeMet or Met. Values are represented as the mean ± the standard deviation (*n* = 5). Statistically significant differences were considered for treatments marked with *(*p* < 0.05)

Cell viability results from MTT assays are shown in Fig. 2. Cisplatin exerted higher cytotoxicity in tumour HeLa cells than in RPTEC/TERT1 cells, being this difference higher at longer incubation times (Fig. 2a, b), confirming the lower resistance of HeLa against this drug [36]. Regarding the employment of Ch-SeNPs, SeMet and Met as coadjuvants, different effects were found. Both SeMet and Met led to decreasing cisplatin-induced nephrotoxicity, resulting in more than a 15% increase in cell viability after 48 h (Fig. 2a, b), in consonance with previous works [8–10, 12]. Meanwhile, no statistically significant changes occurred in HeLa cells when the Pt-drug was co-administered with these compounds (Fig. 2a, b). This confirms their nephroprotective potential, as they exerted protection to renal cells without significantly interfering with the antineoplastic activity of cisplatin.

On the other hand, the viability of RPTEC/TERT1 did not show significant differences whether they were treated with the Pt-drug alone or combined with Ch-SeNPs (Fig. 2a, b). Therefore, although Ch-SeNPs were found to be innocuous for RPTEC/TERT1 (data not shown), they seemed not to exert a direct nephroprotection when co-administrated with cisplatin, in contrast to SeMet and Met. However, the administration of cisplatin and Ch-SeNPs increased the cytotoxicity in HeLa (Fig. 2a, b), demonstrating an enhancement of the anticancer effect of cisplatin. Such an aspect, along with the absence of higher toxicity for kidney cells, may suggest that this combined treatment could allow the use of lower doses of the Pt-drug without losing antitumour efficacy. Considering this, the risk of kidney damage would be diminished; thus, Ch-SeNPs could also be indirectly useful for renoprotection.

### Optimization of working conditions for scICP-MS analysis

A scICP-MS method was optimised to investigate the influence of the different potential nephroprotectors on cisplatin internalisation (referred to as Pt content) in combined treatments. A flow rate of 10 µL min^−1^ and a measurement duration of 100 s were applied in all cases, while different dwell times were evaluated. Cell pellets were re-suspended in ultrapure water (instead of PBS or other buffers or saline solutions) and immediately analysed by scIPC-MS for minimising osmotic stress. Supplementary information (Figures S2, S3) shows that cell integrity is maintained with this procedure. This is in agreement with the results reported by Meyer et al. on scICP-MS analysis of As uptake by human cell lines [20].

After this, it was necessary to find cell concentrations in the resulting suspensions that would provide enough single cell events to obtain a representative sample distribution of the intracellular Pt content. At the same time, highly concentrated suspensions had to be avoided to minimise the simultaneous detection of more than one cell as well as possible deposits along the system (tubes, sample loop, injector, nebuliser, etc.). A possible overestimation of Pt mass in cells due to incomplete removal of the initial cisplatin-containing culture medium from the pellet was also considered. Therefore, cell suspensions were subjected to different dilution factors and washing steps in order to obtain the best results. Analogous Se analyses were also performed to extend this study to the cellular uptake of SeNPs and SeMet.

#### Dwell time and dilution of cell suspensions

Initial tests were performed using different dilutions of RPTEC/TERT1 co-incubated with cisplatin and Ch-SeNPs for 24 h (initial concentration of 5 × 10^6^ cells mL^−1^ after pellet resuspension) to obtain information on Pt and Se from the same sample. Figures S4 and S5 show images of the recorded real-time signal (time scan), including zooms of individual cell pulses to visualise their duration, and the resulting histograms of mass distribution after data processing. Time scans measured for control cells are also shown (Fig. S4, a and S5, a), with no significant signals from either Pt or Se, in contrast to the high number of pulses observed for cells exposed to cisplatin and Ch-SeNPs (Fig. S4, b and S5, b).

First, we evaluated the effect of the dwell time on the TRA measurements after 1:100 dilution of the initial cell suspension, testing four values (50, 100, 150 and 200 µs) (Fig. S6). No significant changes were found in the number of events detected (Fig. S6, a, c) or in the mean mass of Pt and Se per cell (Fig. S6, b, d). These findings are in accordance to the pulse duration observed for each single cell event, which was not lower than 600–800 µs (Fig. S4, c and S5, c). Considering that no settling time was applied in these measurements (i.e., all events occurring along the analysis time, 100 s, were recorded), the dwell times evaluated were shown to allow full integration of the signal pulses and also a non-significant risk of cell coincidence in the same event. Therefore, further analyses were decided to be performed at a fixed dwell time of 100 µs.

**Fig. 3:**
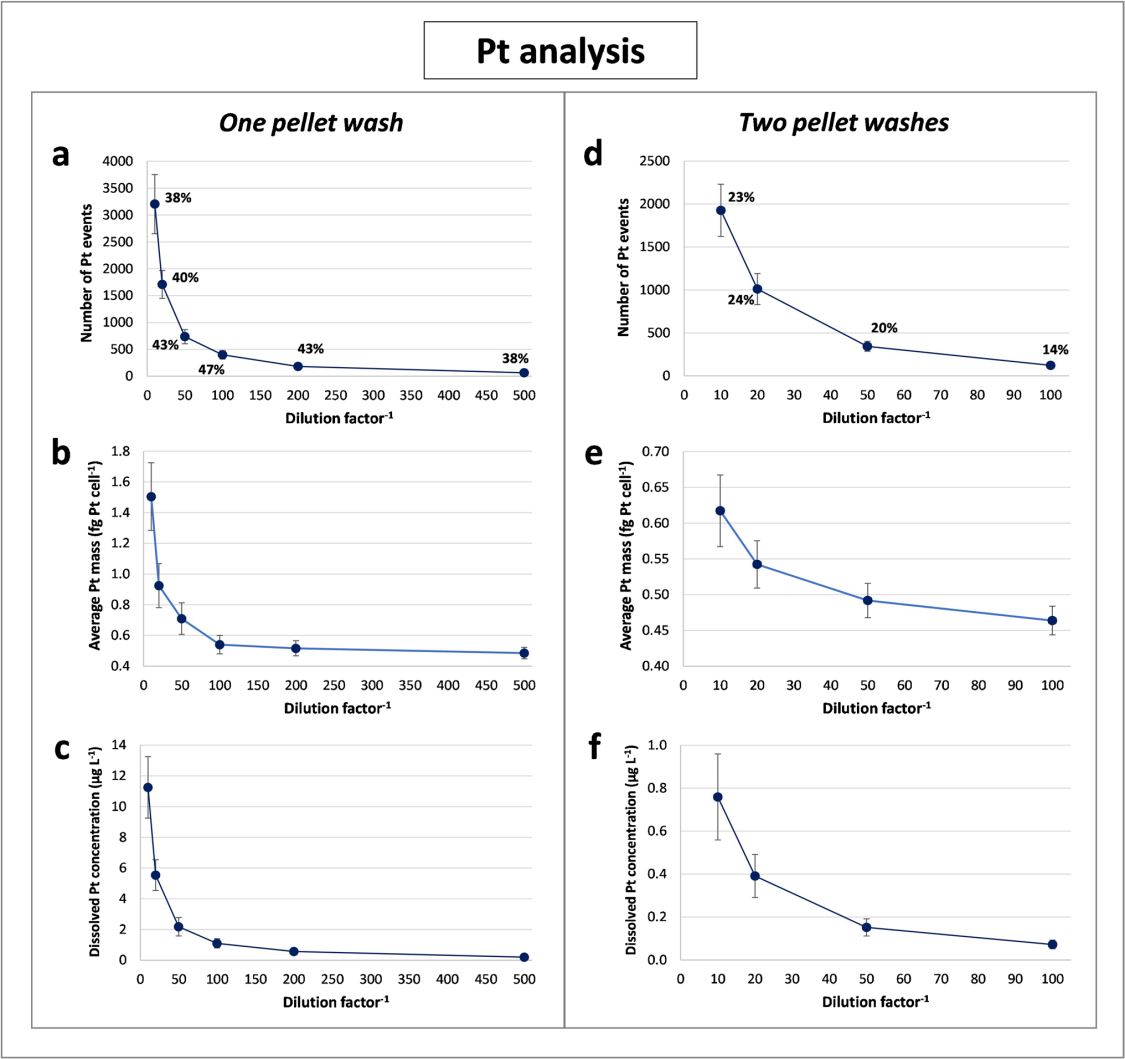
Plots corresponding to scICP-MS measurements of ^195^Pt in RPTEC/TERT1 exposed to cisplatin and Ch-SeNPs for 24 h after one (**a**, **b**, **c**) or two pellet washes (**d**, **e**, **f**): variation of the number of single cell events (**a**, **d**), the average element content per cell (**b**, **e**) and the extracellular element concentration (**c**, **f**) with the dilution factor of the cell suspensions. All the results are presented as the mean ± the standard deviation (*n* = 3). The ratios of detected single cell events to expected events (%) for each dilution are shown in plots **a** and **d**

**Fig. 4:**
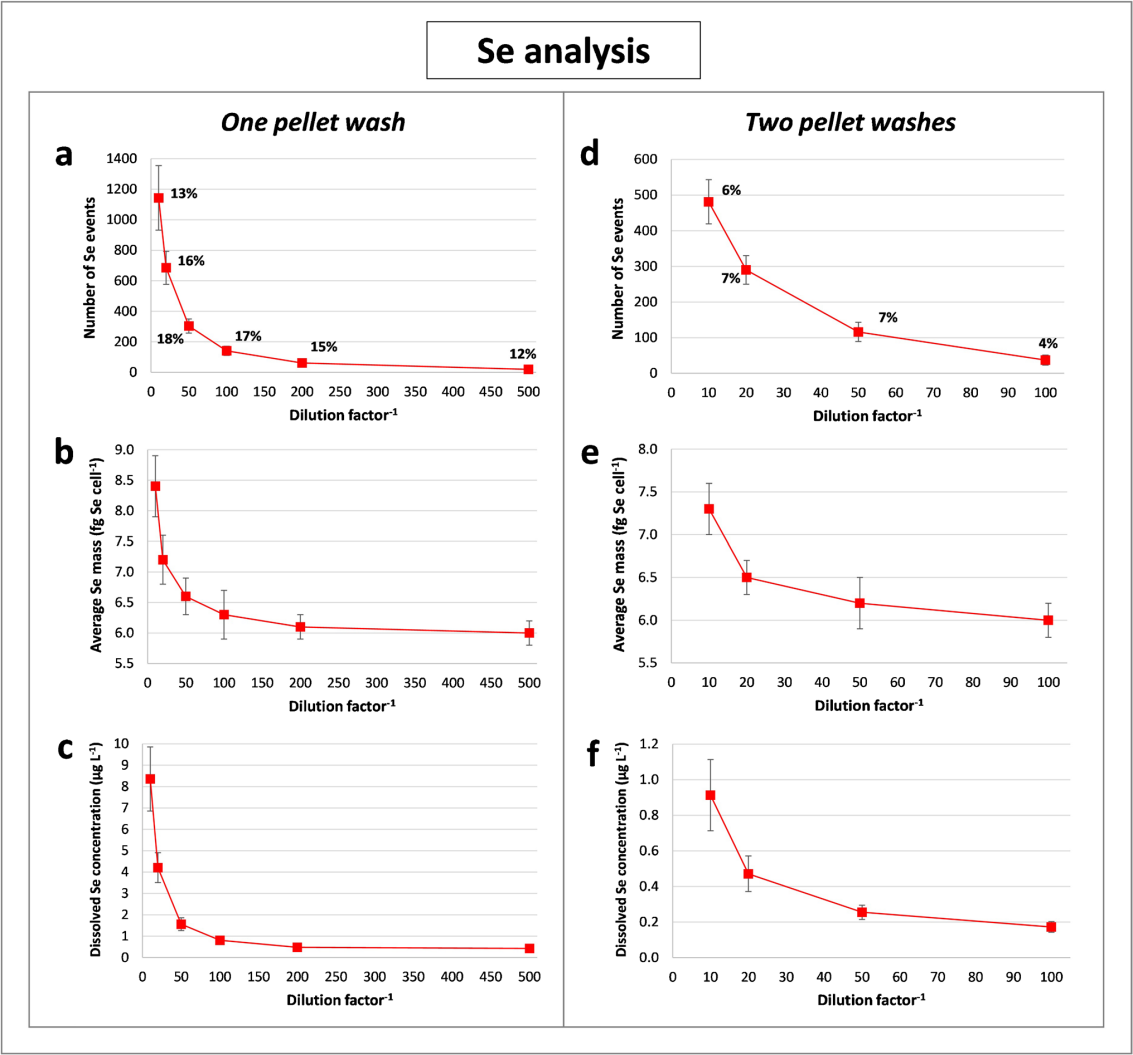
Plots corresponding to scICP-MS measurements of ^78^Se in RPTEC/TERT1 exposed to cisplatin and Ch-SeNPs for 24 h after one (**a**, **b**, **c**) or two pellet washes (**d**, **e**, **f**): variation of the number of single cell events (**a**, **d**), the average element content per cell (**b**, **e**) and the extracellular element concentration (**c**, **f**) with the dilution factor of the cell suspensions. All the results are presented as the mean ± the standard deviation (*n* = 3). The ratios of detected single cell events to expected events (%) for each dilution are shown in plots **a** and **d**

After this, the influence of the dilution factor on the estimation of the mean intracellular content of Pt and Se, the number of single cell events and the extracellular concentration of both elements was studied, as can be seen in Figs. 3 and 4. The number of ^195^Pt events was found to be 38–47% of the total cell events expected in each measurement (calculated from the cell concentration in each dilution, sample flow rate and analysis time), almost three times higher than for ^78^Se (12–18%) (Fig. 3a and Fig. 4a), suggesting an easier uptake of the Pt-drug. The proportion of Pt events—that is, the ratio of detected Pt events to the number of total events expected—are comparable to those obtained for the certified AuNPs (> 40%). As the transport of cells is unlikely to be easier than for nanoparticles, it could then be assumed that no more than 40% of the aspirated cells would reach the plasma. Considering that the transport efficiencies for human cell lines reported in the literature are often lower than 40% [19, 22, 24], we assumed that the percentage found here for Pt may be sufficiently representative of the cell transport efficiency. Pt was selected for this purpose as it offered a higher sensitivity and less interference than endogenous elements such as Cu, Zn or Fe, providing higher reproducibility of the measurements in ICP-MS analysis.

Despite the washing steps done to cells, a high concentration of dissolved Pt was found (around 11 µg L^−1^ after a 1:10 dilution) but also in the case of Se (close to 8 µg L^−1^ for the same dilution) (Fig. 3c and Fig. 4c), which could be related to residual selenite from the synthesis of Ch-SeNPs or selenite released by cells after their exposure to Ch-SeNPs. As expected, this extracellular concentration of both elements was decreased with increasing dilution of the cell suspensions, as also occurred with the number of single cell events (Fig. 3a, c and Fig. 4a, c). However, sample dilution also reduced the average intracellular mass of each element, although this value should remain unchanged regardless of the number of cells analysed (Fig. 3b and Fig. 4b). The effect was particularly strong for Pt, which decreased by more than 100% when comparing the mean values with 1:10 and 1:100 dilutions of the cell suspension (approximately 1.5 and 0.6 fg Pt cell^−1^, respectively), whereas the reduction for Se was lower than 25% (approximately 8.4 and 6.3 fg Se cell^−1^, respectively). No significant changes were found at dilution factors higher than 1:100 when the dissolved Pt concentration was reduced below 2 µg L^−1^ (Fig. 3b, c). Although the decrease in intracellular Pt mass might have been attributed to cell disruption due to osmotic stress with increasing dilution in water, this was discarded as the reduction in the Se content (Fig. 4b) was much lower than for Pt, and the percentage of detected events of Pt and Se did not show significant variations as the cell concentration in the samples decreased (Fig. 3a and Fig. 4a). The small changes observed in the ratio between detected and expected events, regardless of the dilution factor applied (Fig. 3a and Fig. 4a), allowed to exclude the risk of a false mass determination due to the occurrence of pulses corresponding to more than one cell.

Considering the above, the variation of the intracellular Pt content seems to be related to an overestimation effect produced by the signal of dissolved Pt itself. As this value increases, the signals corresponding to cells with lower accumulation are less likely to exceed the intensity threshold established by the iterative calculations to find the real single cell events (pulse intensity > mean intensity in the sample + *k* · sd (standard deviation), where *k* is equal to or greater than 3), resulting in an increased mean mass of the cell population. Referring to Fig. 3b and c, the concentration of extracellular Pt must be reduced below 2 µg L^−1^ to minimise its influence on the average content inside the cells. On the other hand, the reduced Pt mass in cells with increasing sample dilution could also be related to a progressive removal of cisplatin, not internalised but bound to the cell surface during the resuspension cycles of the cell samples. Currently, scICP-MS cannot distinguish the signal of the analyte actually inside the cell from that of the analyte adsorbed to the cell membrane, so both contributions are included in the intracellular mass determined. This risk seemed to be less important for Ch-SeNPs, since TEM images showed that the vast majority were internalised by the cells and only a very small amount was located on their surface (previous section). Thereby, to minimise the undesirable contribution of both dissolved elements and the analytes bound to the cell membrane in the estimation of intracellular mass values, high dilutions are necessary.

#### Number of cell pellet washes

In parallel, the same study was carried out after applying an additional wash with PBS to the collected pellet (therefore, a total of two washes), considering that it could be efficient enough to remove most of the non-internalised particles, as previously reported for TiO_2_NPs and AgNPs association with cell lines from aquaculture species [23]. In this case, the number of single cell events registered for Pt and Se—expressed as both absolute values and ratios of detected to expected events—was almost half the value previously observed (Fig. 3d and Fig. 4d), mainly due to a partial loss of cells when discarding the supernatant from the second wash. The average content of Pt and Se per cell also showed a significant decrease, as occurred with increasing dilutions (Fig. 3e and Fig. 4e). On the other hand, the extracellular concentration of both elements was highly decreased (Fig. 3f and Fig. 4f), and a lower dilution factor was required to obtain a reliable value of the intracellular mass of Pt and Se (taking as reference the minimum dilution factor where no further significant decreases in these contents were found) (Fig. 3e and Fig. 4e). Again, this was especially relevant for Pt, with a variation in the mean mass in cells close to 25% between the 1:10 and 1:100 dilutions, and a concentration of dissolved element lower than 0.8 µg L^−1^ even for the 1:10 dilution, in contrast to the equivalent results without the extra wash mentioned above (more than 100% and 11 µg L^−1^, respectively). Therefore, both the higher dilution of the cell suspensions and the addition of an extra washing step of the pellet can provide similar results if enough cells are present in the samples prior to their analysis. Thus, the combination of both methods was shown to be preferable to multiple washing or over-dilution.

### Comparison between conventional setting conditions (NexION 300X) and highly efficient sample introduction systems (NexION 2000) for scICP-MS

To validate the previous results, scICP-MS analyses were also performed with no special settings for working with intact cells (conventional sample introduction system and cyclonic chamber in a NexION 300X), but also operating in TRA mode. Figures S4 and S5 show the histograms of mass distribution obtained when using a specialised autosampler, high-efficiency introduction system (CytoNeb) and Asperon spray chamber (Fig. S4, d and S5, d) and conventional ICP-MS (Fig. S4, e and S5, e) for renal cells treated with cisplatin and Ch-SeNPs. The uptake pattern for Pt and Se was similar in both cases, with no significant differences between the mean masses estimated per cell: 0.74 fg Pt cell^−1^ and 6.4 fg Se cell^−1^ with special single cell setting conditions and 0.64 fg Pt cell^−1^ and 5.5 fg Se cell^−1^ with conventional conditions. These results are in consonance with the not-quite-different LOD achieved with the two models (see the ‘Materials and methods’ section). Although the sample flow rate used with the NexION 300X (100 µL min^−1^) was ten times higher than with the NexION 2000, the dwell time chosen (100 µs) avoided the risk of mass overestimation caused by the simultaneous detection of multiple cells. The number of single cell events detected for both Pt and Se was more than 40% higher with NexION 300X than with NexION 2000, as expected due to the higher flux used in the first case. However, the ratio of detected to expected events was much lower (< 4% and < 2% for Pt and Se, respectively) due to the less efficient sample introduction system of the NexION 300X, which was not specifically designed for single cell analysis. On the contrary, cell integrity was confirmed to be unaffected by this lower cell transport efficiency, as both settings gave similar results for the intracellular content of Pt and Se.

### Comparison of scICP-MS and bulk ICP-MS analysis after acid digestion of cells

The intracellular content of Pt and Se in RPTEC/TERT1 cultures with cisplatin and Ch-SeNPs was also determined by the conventional method based on bulk analysis of digested pellets, studying as well the effect of cell pellet washing (Figure S7). A significant decrease in the mean mass was shown when increasing the number of washing cycles from one to four (Fig. S7, a, b), confirming the overestimation effect due to the extracellular presence of both analytes, as observed during scICP-MS analysis. The Pt and Se contents obtained (Fig. S7, a, b) were much higher than those determined by scICP-MS (Fig. 3b, e and Fig. 4b, e), which could be related to the inability of bulk analysis to discern whether the analyte is inside or outside the cells, in contrast to TRA mode. To evaluate this, a possible contribution of dissolved Pt to the average intracellular mass of this element was estimated with the data registered by scICP-MS. In the case of 1:10 dilutions of cells exposed to cisplatin and Ch-SeNPs (Fig. 3c, f), the concentration of extracellular Pt before dilution would be 110 and 8 µg L^−1^ after one and two pellet washes, respectively. Considering the volume of the initial suspension (1 mL) and the total number of cells (approximately 5 × 10^6^), the dissolved Pt would correspond to 22 and 1.6 fg Pt cell^−1^, respectively. This contrasts with the lower intracellular content provided by scICP-MS (1.5 and 0.6 fg Pt cell^−1^) (Fig. 3b, e), demonstrating how the presence of dissolved Pt can severely bias the values obtained from bulk analysis if the cell pellets are not sufficiently washed. Nevertheless, even after a total of four washing cycles, the values of Pt and Se after acid digestion (8 fg Pt cell^−1^ and 37 fg Se cell^−1^) (Fig. S7, a, b) were still almost 16-fold and sixfold higher, respectively, than those resulting from the time-resolved analysis of intact cells (Fig. 3b, e and Fig. 4b, e). In this case, the pellets were expected to be sufficiently washed to have a negligible extracellular contribution of both elements. Therefore, incomplete removal of residual cisplatin and SeNPs not internalised by cells does not seem to be the only factor explaining these differences.

The discrepancy between the two methods has also been observed in previous works where scICP-MS results could be more than 30 times lower than bulk analysis [21, 23]. Some authors have suggested that this could be due to detector saturation during scICP-MS measurements of cells containing a high number of nanoparticles [21, 23]. However, the same underestimation was observed in the current investigation for the intracellular content of a molecular compound such as cisplatin. This would suggest that the detection efficiency for undigested cells could be compromised, regardless of whether the internalised analyte is in dissolved or colloidal form, at least if its concentration is above a certain limit. The bias could also arise from the use of dissolved standards of Pt and Se for calibrations assuming a similar behaviour inside the ICP-MS plasma as in the case of cells. Despite the correction with the transport efficiency determined with the AuNPs standard, a hypothetical less efficient atomisation and ionisation for intact cells is not taken into account, which could lead to an underestimation of the actual intracellular mass. For this reason, one-point calibrations using PtNPs (40 nm) and SeNPs (120 nm) were also tested to quantify the content of Pt and Se per cell, taking advantage of the higher physical similarity between cells and nanoparticles instead of dissolved standards. Although no significant differences were found (data not shown) with respect to the ionic calibration, it would be necessary to also assess whether the atomisation rates of cells and nanoparticles are indeed comparable to avoid erroneous estimations. On the other hand, the accuracy of the cell count in the original sample could also be a critical factor for this disparity, as it affects the mean mass calculated by the conventional method based on acid digestion, but not in the case of scICP-MS. Thus, considering the differences that could be found in the average intracellular contents quantified by both methodologies, their combined use for validation purposes may be preferable to compare uptake trends in the different treatments instead of absolute values.

### Determination of cellular uptake of Pt and Se by scICP-MS

#### Internalisation of dissolved and colloidal forms of Se

**Fig. 5:**
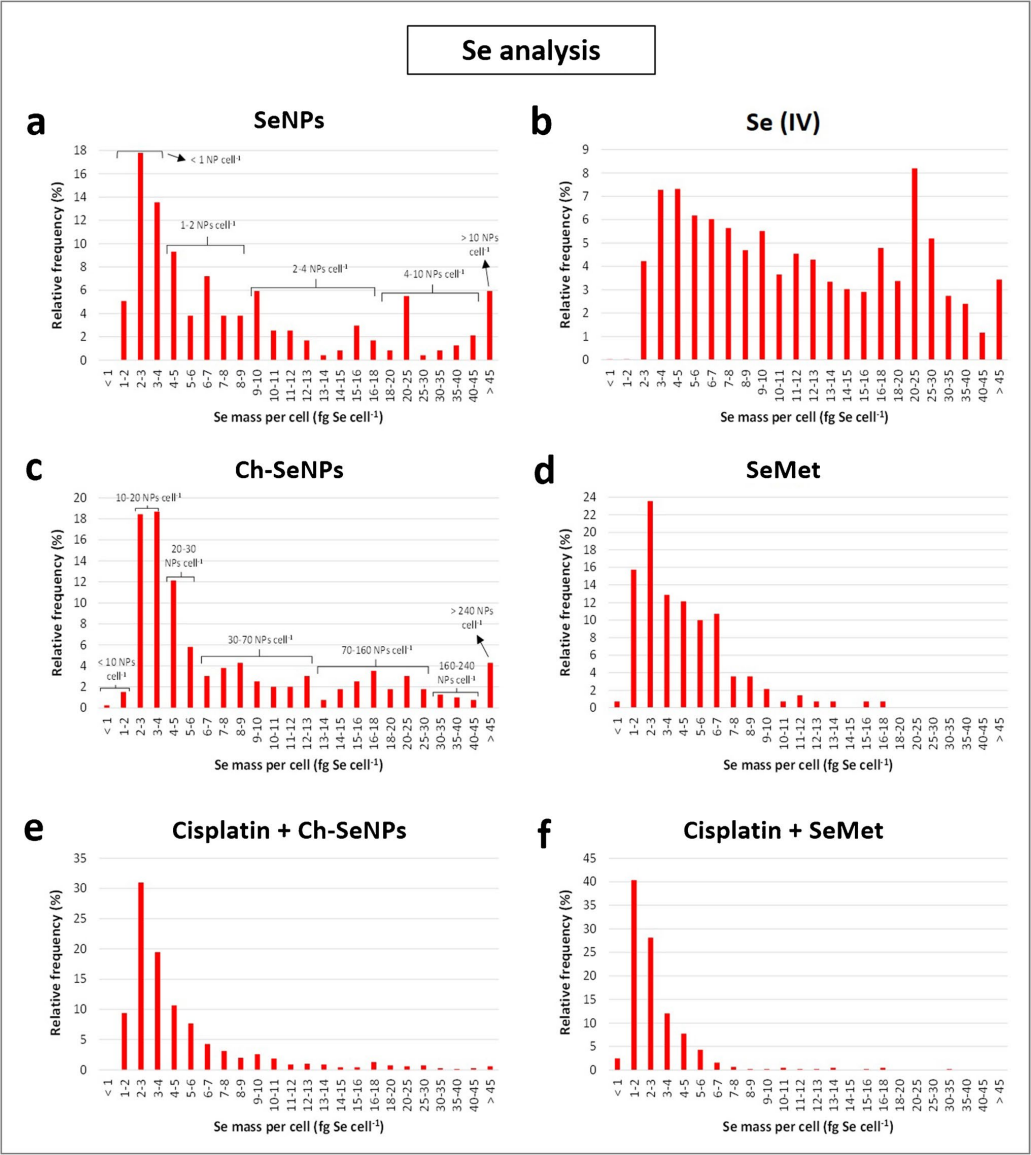
Histograms of the mass distribution obtained by scICP-MS for the intracellular content of Se corresponding to 24 h cultures of RPTEC/TERT1 with uncapped SeNPs (**a**), selenite (**b**), Ch-SeNPs (**c**), SeMet (**d**), cisplatin + Ch-SeNPs (**e**) and cisplatin + SeMet (**f**). The estimated numbers of SeNPs per cell were indicated for cultures with uncapped SeNPs (**a**) and Ch-SeNPs (**c**)

Before studying the influence of renoprotectors on the internalisation of cisplatin by cells, the potential of scICP-MS to analyse the uptake of different chemical forms of Se (Ch-SeNPs, SeNPs, SeMet and Se(IV)) after 24 h incubation was first evaluated. Following the results of the optimisation tests, cell pellets were washed twice with PBS after collection, dissolved in 1 mL of ultrapure water and then diluted 1:20 to 1:50 with the same solvent to have a similar number of cells in all samples before their analysis. Figure 5 shows the different Se mass histograms obtained for RPTEC/TERT1.

The Se profiles of cells treated with Ch-SeNPs and uncapped SeNPs appeared to be similar (Fig. 5a, c), with mean Se contents not significantly different (10.2 and 12.3 fg Se cell^−1^, respectively). However, taking into account the mean NP diameters revealed by TEM (42 and 120 nm, respectively) (Fig. S1, d, h) and their corresponding masses (close to 0.2 and 4.3 fg Se, respectively), the degree of internalisation was shown to be much higher for Ch-SeNPs (75% of cells containing 10–70 NPs cell^−1^ and more than 20% with 70–240 NPs cell^−1^) than for uncapped SeNPs (about 90% of cells internalising less than 10 NPs cell^−1^) (Fig. 5a, c). In the second case, a significant decrease in the number of Se events recorded (280 events) was observed compared to the incubation with Ch-SeNPs (396 events), confirming the more efficient uptake of the smallest NPs. Nearly 35% of the Se events detected for incubation with uncapped SeNPs corresponded to masses not higher than that calculated for one of these SeNPs (120 nm, about 4.3 fg Se). This could be related to the cellular internalisation of SeNPs smaller than 120 nm or Se(IV) ions (remaining from NP synthesis or resulting from oxidised SeNPs), or a partial release of Se by cells after internalising SeNPs. Another cause could be the appearance of pulses corresponding to single SeNPs that were outside the cells, since current scICP-MS analysis is not able to distinguish whether a pulse corresponds to a single extracellular NP or to a NP internalised by a single cell. Therefore, in the particular case of cells treated with bare SeNPs (Fig. 5a), there were a high proportion of Se pulses that could not correspond to the Se contained by single cells but to not internalised SeNPs. Nevertheless, nearly 50% of the events had an intensity higher than that shown by two or more uncapped SeNPs. As negligible occurrence of multiple events due to the simultaneous detection of more than one particle or cell was observed with our instrumental conditions (10 µL min^−1^ as sample flow, and 100 µs as dwell time), we can assume that at least half of the Se events found actually corresponded to the content of Se in single cells. On the contrary, all the Se pulses registered for cultures with Ch-SeNPs (Fig. 5c) can be assessed to be unequivocally produced by the mass of Se contained by cells. We concluded this after verifying that the signal of single Ch-SeNPs (42 nm) not internalised by cells was not distinguishable from the corresponding to background or dissolved Se due to the high LOD of Se.

Regarding the use of molecular selenocompounds (Se(IV) and SeMet), important changes were observed compared to colloidal Se (Fig. 5a–d). Exposure to selenite led to a slightly higher Se content than those obtained with SeNPs (mean value of 14.2 fg Se cell^−1^) and to a higher number of cells internalising the selenocompound (1161 Se events, about three times higher than with SeNPs), which contrasts with the results obtained for SeMet (5.0 fg Se cell^−1^, 352 Se events). The uptake of Se(IV) seems to be the most favourable, especially compared to other molecular forms such as SeMet. However, this process can also be very heterogeneous among the cells of a sample, as shown by the strong polydispersity in the histogram of the mass distribution corresponding to selenite (Fig. 5b).

#### Effect of the co-administration of potential nephroprotectors on cisplatin internalisation

**Fig. 6:**
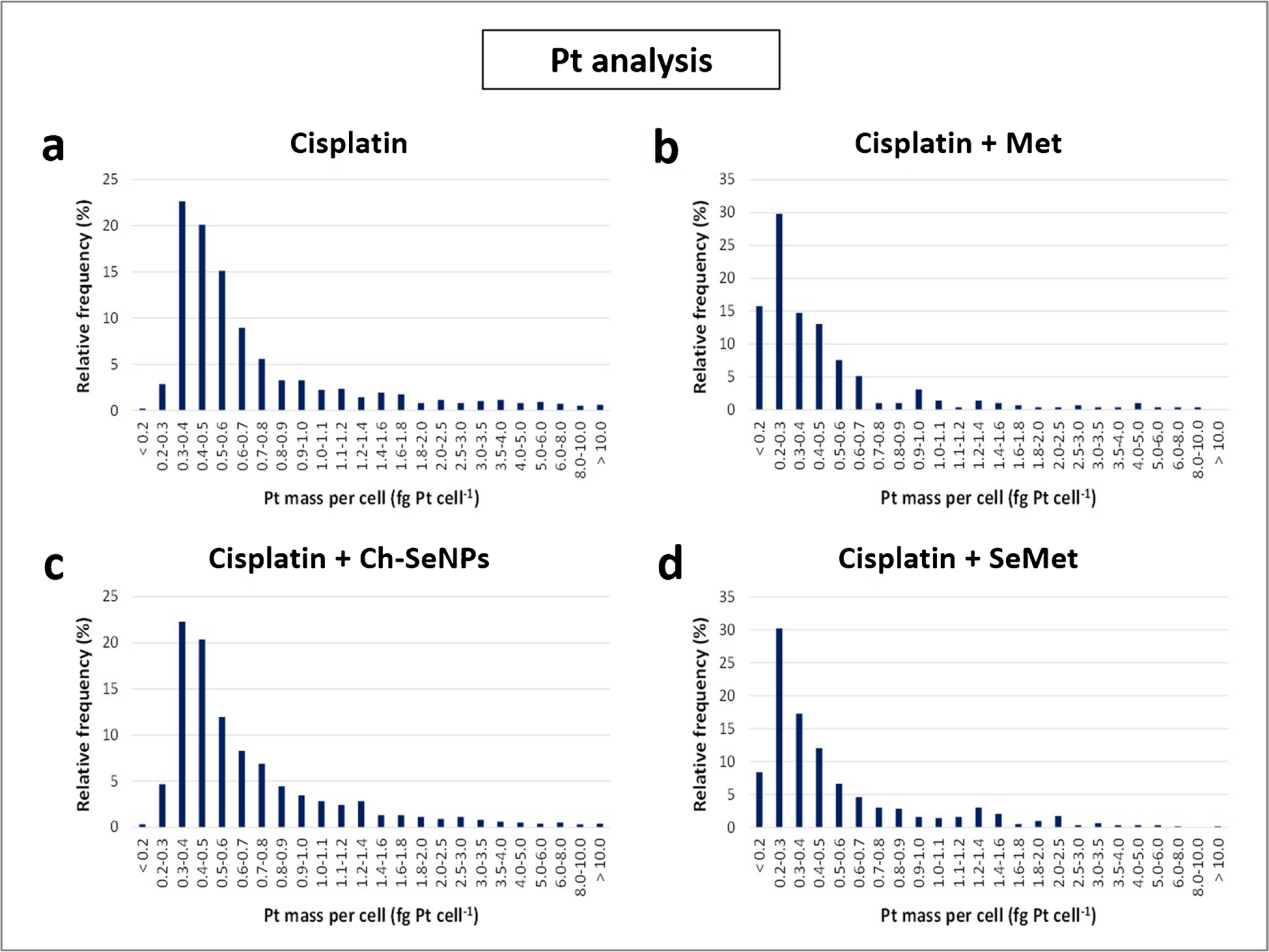
Histograms of the mass distribution obtained by scICP-MS for the intracellular content of Pt corresponding to 24-h cultures of RPTEC/TERT1 with cisplatin (**a**), cisplatin + Met (**b**), cisplatin + Ch-SeNPs (**c**) and cisplatin + SeMet (**d**)

**Fig. 7:**
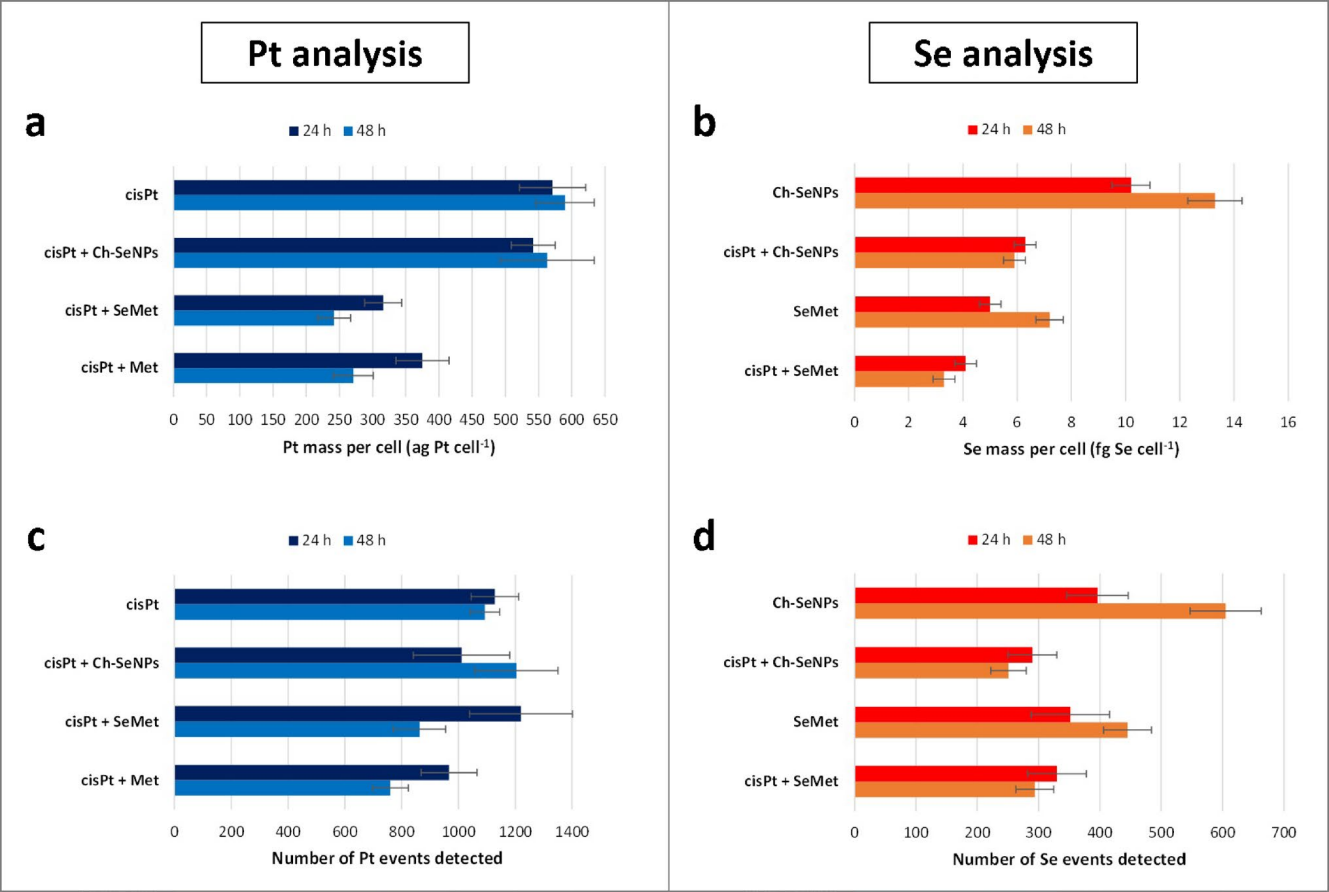
Graphs comparing the intracellular contents (**a**, **b**) and the number of registered single cell events (**c**, **d**) during scICP-MS measurements of ^195^Pt (**a**, **c**) and.^78^Se (**b**, **d**) in RPTEC/TERT1 exposed for 24 and 48 h to cisplatin, Ch-SeNPs, SeMet, cisplatin + Ch-SeNPs, cisplatin + SeMet and cisplatin + Met. All the results are presented as the mean value ± the standard deviation (*n* = 3)

Analysis of the cellular uptake of cisplatin in the presence of the three coadjuvants studied (Ch-SeNPs, SeMet, and Met) was performed after 24 and 48 h of exposure. The influence of the Pt-drug on the internalisation of Ch-SeNPs and SeMet was also evaluated. The results corresponding to the cultures with renal cells can be seen in Figs. 6 and 7. As indicated in the previous section on the initial optimisation of the analyses, no relevant signals of Pt or Se were found for control cells, and Pt and Se could only be determined in cultures with cisplatin or selenocompounds, respectively. The profiles of the Pt histograms of the 24 h incubations (Fig. 6a–d) were similar in most cases, as could be expected considering that cisplatin was the only Pt-compound involved. However, the mass distribution was slightly broader when cisplatin was used alone (Fig. 6a) or in the presence of Ch-SeNPs (Fig. 6c) compared to the other treatments (Fig. 6b, d), showing a higher Pt uptake in the first two cases. This fact was confirmed by the average intracellular values (Fig. 7a and Table S2, a), while no significant differences were detected after 24 h exposure to cisplatin alone or co-administered with Ch-SeNPs, a clear decrease occurred with SeMet (35%) and Met (31%) as coadjuvants. These changes were even more obvious in 48-h cultures, both in the amount of internalised Pt per cell (decreases of 55% and 46%, respectively) (Fig. 7a and Table S2, a) and in the number of Pt events registered, which represents the number of cells internalising the Pt-drug (decreases of 21% and 30%, respectively) (Fig. 7c). The reduction in the intracellular Pt levels in the presence of SeMet and Met can be correlated with the lower cisplatin-induced toxicity observed by MTT assays (Fig. 2a, b), reinforcing the previous reports about their renoprotective potential [8–10, 12]. On the other hand, the unchanged cellular uptake of Pt regardless of cisplatin was administered alone or combined with Ch-SeNPs may discard that they could protect kidney cells by reducing drug accumulation on them. However, this aspect is confirmed to be positive to preserve the Pt-drug anticancer efficacy, attending to cell viability results in HeLa cells (Fig. 2a, b).

Regarding Se measurements, the uptake rate of the two Se compounds in the absence of cisplatin showed an increase over time, being higher with Ch-SeNPs (Fig. 7b, d). In both cases, co-treatment with the Pt-drug caused a significant reduction in the accumulation of Se per cell (at least 20% and 50% after 24 and 48 h, respectively) (Fig. 7b and Table S2, b) and in the number of cells internalising Ch-SeNPs or SeMet, especially after 48 h (58% and 34%, respectively) (Fig. 7d), demonstrating a cisplatin-induced impairment of cellular endocytosis and transporters involved in the internalisation of these selenospecies. Similar results were found for HeLa in terms of cisplatin uptake (Table S2, a). However, the Pt-drug caused a higher impact on this cell line related to Se accumulation, not only in the intracellular content (Table S2, b) but also in the number of single cell events of Se, which decreased more than 75% from the first 24 h of incubation (data not shown). This may agree with the lower resistance of the tumour cells against the Pt-drug, as confirmed previously by MTT assays (Fig. 2a, b). Cell viability results also demonstrated that the decreased uptake of SeMet and Ch-SeNPs triggered by cisplatin did not prevent their biological effects, in terms of enhanced nephroprotection for SeMet or enhanced antitumoricity for Ch-SeNPs (Fig. 2a, b).

The different cultures were also analysed after acid digestion of the pellets, providing mean values of intracellular Pt and Se (data not shown) much higher than those corresponding to scICP-MS. Nevertheless, the same trends related to cisplatin accumulation in the presence of the protectors were observed, with no influence of Ch-SeNPs co-administration, while a high Pt decrease was induced by SeMet and Met (Table S2, a). Although higher differences between the two techniques were found in the case of Se, digestion-based measurements also confirmed the reduced internalisation of Ch-SeNPs and SeMet when co-treated with the Pt-drug (Table S2, b).

## Conclusions

An optimised scICP-MS method has successfully been employed for the evaluation of the internalisation of cisplatin in co-treatments with Ch-SeNPs, SeMet and Met as potential nephroprotectors in both renal and tumour cells. The uptake of the different selenocompounds was also studied. The results showed a reduced accumulation of cisplatin in the presence of SeMet or Met, which may be related to the protection that both exerted on renal cells against the drug. Ch-SeNPs had no effect on cisplatin uptake, but their use as a coadjuvant led to a higher antineoplastic efficacy, giving the chance to reduce the Pt-drug dose and thus, indirectly, kidney toxicity. The intracellular contents of both Ch-SeNPs and SeMet decreased when co-administered with cisplatin, although this did not impair their pharmacological effects. Measurements using two ICP-MS settings either with a conventional introduction system or specifically configured for single cell work were done to validate these results. Besides, the trends observed regarding the changes in the levels of internalised Pt and Se were confirmed with analysis after cell digestion. Unlike conventional bulk measurements, scICP-MS provided not only mean values of the intracellular contents of Pt and Se but also a profile of mass distribution within the cell population and the number of cells internalising cisplatin and selenocompounds, demonstrating its high versatility and potential for cellular studies in biomedical research.

## Supplementary Information

Below is the link to the electronic supplementary material.

Supplementary File 1 (DOCX 5.39 MB)

## Data Availability

Data is provided within the manuscript or supplementary information files.
