# Supplementary material for: Single cell ICP-MS for the assessment of potential nephroprotectors against cisplatin
Source: Mikrochim Acta. 2025 Jul 23;192(8):514. doi: 10.1007/s00604-025-07383-8 (PMC12287176; doi:10.1007/s00604-025-07383-8)
Supplement: Supplementary file 1 — (DOCX 5.39 MB) [file 604_2025_7383_MOESM1_ESM.docx]

**SUPPLEMENTARY INFORMATION**

**Single cell ICP-MS for the assessment of potential nephroprotectors against cisplatin**

*Alejandro Iglesias-Jiménez^a^, Gema Artiaga^a^, Estefanía Moreno-Gordaliza^a^, Pilar Bermejo-Barrera^b^, Antonio Moreda-Piñeiro^b^, Mª Milagros Gómez-Gómez^a*^*

^a^Department of Analytical Chemistry, Faculty of Chemical Sciences, Universidad Complutense de Madrid, Avenida Complutense s/n, 28040 Madrid, Spain

^b^Group of Trace Elements, Spectroscopy and Speciation (GETEE), Institute of Materials iMATUS, Department of Analytical Chemistry, Nutrition and Bromatology. Faculty of Chemistry, University of Santiago de Compostela, Avenida das Ciencias, s/n 15782 Santiago de Compostela, Spain

E-mail address: alejandroiglesias@ucm.es (Alejandro Iglesias-Jiménez), gmaayuso@ucm.es (Gema Artiaga), emorenog@ucm.es (Estefanía Moreno-Gordaliza), pilar.bermejo@usc.es (Pilar Bermejo-Barrera), antonio.moreda@usc.es (Antonio Moreda-Piñeiro) and mmgomez@ucm.es (Mª Milagros Gómez-Gómez)

*Corresponding author

**Figure S1.** TEM-EDS analyses of uncapped SeNPs (a, b, c, d) and Ch-SeNPs (e, f, g, h): TEM images (a, b, e, f); EDS spectra (c, g); and size distribution histograms (d, h).

**Evaluation of the effect of ultrapure water for the resuspension and dilution of cell samples before scICP-MS analysis**

First, we prepared four replicates of HeLa exposed to either cisplatin (9 mg L^-1^) or Ch-SeNPs (5 mg L^-1^) for 24 hours. Once finished the incubation time, we collected and washed cell pellets as described in the “Collection of pellets after cell incubations” section of the manuscript (inside “Materials and methods”). Then, just before scICP-MS measurements of Pt and Se, two pellets (A, C) were resuspended in PBS (1 mL) and one (B) in Milli-Q water (1 mL). The last pellet (D) was dissolved in a home-made buffer solution (1 mL) that we use for cell total lysis to obtain protein extracts. This allowed us to know the behaviour of actually lysed cells during TRA analysis, being useful for comparison with the other three samples (A, B and C). The solution containing pellet D was sonicated with an ultrasound probe (20 pulses/sample -2 s “on” and 5 s “off”- and 40 % of amplitude) and then ultracentrifuged (13,000 rpm, 4 °C, 10 min), to achieve an effective cell lysis. The resulting pellet after this ultracentrifugation (solid fraction containing lysed cells) was isolated from the supernatant, and resuspended in ultrapure water (1 mL). Finally, the following 1:20 dilutions were done prior to their analysis: using PBS for sample A; and employing Milli-Q water for the other three samples (in the case of sample D, after cell lysis and further pellet resuspension also in Milli-Q water).

As can be seen in **Figure S2**, the Pt profile in the mass distribution histograms was very similar for samples A, B and C (**Fig. S2, a-c**). This also occurred with the mean intracellular Pt content (ranging between 0.80 and 0.94 fg Pt cell^-1^) and the number of single cell events of Pt detected (ranging between 1350 and 1780). The conclusions were the same after the evaluation of Se results (data not shown). In contrast, as expected, cell lysis strongly affected on sample D (**Fig. S2, d**), showing a remarkable different Pt profile, a lower mean intracellular Pt content (0.17 fg Pt cell^-1^) and much more events (9610), in consonance with the formation of different cell fragments. Therefore, our cells did not exhibit evidences of lysis regardless of being dissolved and diluted in PBS or Milli-Q water (samples A, B and C), unless they were submitted to a specific procedure to achieve cell fragmentation (sample D). At the same time, the similar results obtained for samples A (resuspension and dilution in PBS) and C (resuspension in PBS and dilution in ultrapure water) (**Fig. S2, a, c**) suggest that the use of ultrapure water did not produce cell disruption not only during pellet resuspension (as concluded after the comparison between samples A and B (**Fig. S2, a, c**)) but also during further dilution of cell suspensions.

Besides, we evaluated a possible damage on cell morphology due to the employment of Milli-Q water instead of PBS or collisions suffered by cells inside the nebulizer or the spray chamber. For this aim, we collected the liquid waste from the spray chamber drain during the TRA analysis of a suspension of cells in ultrapure water, and then it was transferred to a P100 plate containing fresh culture medium. At the same time, new cells of the same line were seeded in a different P100 plate using fresh medium as well. In this case, cells had no contact with Milli-Q water in order to guarantee their integrity. We observed both samples under an optical microscope, with 10x and 40x magnifications (**Figure S3**), and no apparent differences were found in terms of morphology. The characteristic spherical shape of cells just detached and transferred to a new plate seemed to be preserved after their introduction into the nebulizer (**Fig. S3, a, c**). The fewer number of cells collected from the waste (**Fig. S3, a, c**), compared to the observed in the other plate (**Fig. S3, b, d**), is not related to a possible cell disruption but to the low sample flow and the high cell dilutions used for scICP-MS analysis.

Thus, we believe that our cells did not suffer significant breakage or fragmentation during the measurements, either due to the employment of ultrapure water as solvent or possible collisions inside the nebulizer or the spray chamber. The fact that we resuspended and diluted cell pellets in Milli-Q water just before the ICP-MS measurements could decrease the risk of cell lysis produced by osmotic stress, compared to cells with longer contact with this solvent. On the other hand, we used a high-efficiency introduction system specifically designed for scICP-MS analysis of intact cells (including CytoNeb nebulizer and Asperon spray chamber), so it seems unlikely that we had problems related to cell breakage during sample introduction.

**Figure S2.** Histograms of the mass distribution obtained by scICP-MS for the intracellular content of Pt corresponding to 24-hour cultures of HeLa co-administered with cisplatin and Ch-SeNPs: using PBS for both pellet resuspension and further 1:20 dilution (a); using Milli-Q water for both pellet resuspension and further 1:20 dilution (b); using PBS for pellet resuspension and Milli-Q water for further 1:20 dilution (c); and using lysis buffer for resuspension and cell rupture assisted by ultrasonication and ultracentrifugation, prior to the final 1:20 dilution in Milli-Q water of the lysed cells.


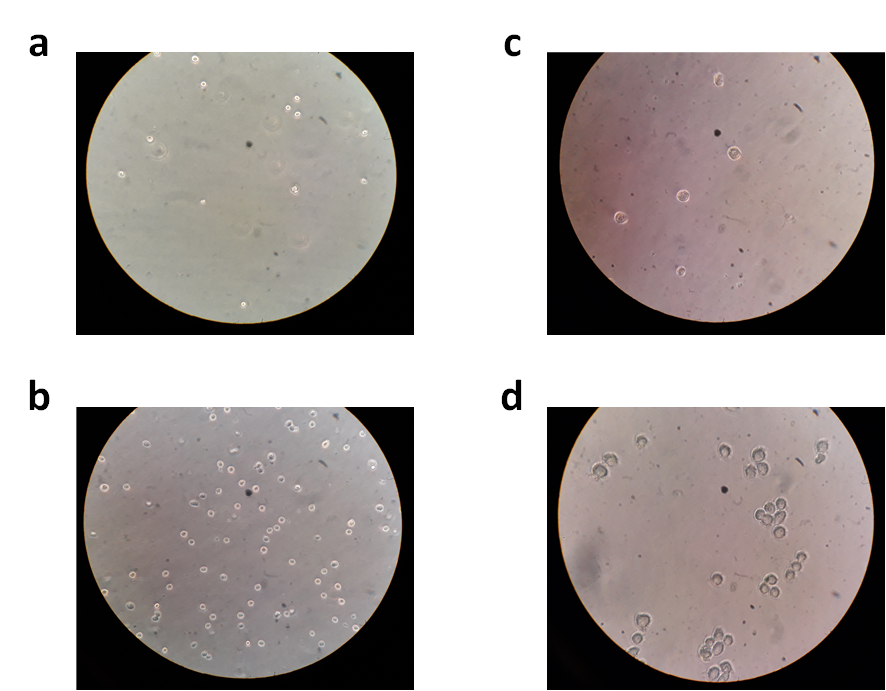


**Figure S3.** Inverted microscope images at 10x (a, b) and 40x (c, d) magnification obtained from 24-hour cultures of HeLa treated with cisplatin and Ch-SeNPs: after collected from the waste of the spray chamber drain during scICP-MS measurements and re-seeded in a P100 plate with fresh medium (a, c); and after a direct seed in a P100 plate with fresh medium without having been previously introduced in the ICP-MS (b, d). The cells shown in Fig. S3, a. c were initially resuspended and diluted in Milli-Q water prior to their analysis.

**Figure S4.** Data obtained after scICP-MS analyses of ^195^Pt in RPTEC/TERT1: time scans for 1:50 dilutions of control cells (a) and cells incubated with cisplatin and Ch-SeNPs for 24 h (b), including an amplified single cell pulse for the second case (c); and histograms of mass distribution (d, e) for cells co-treated with cisplatin and Ch-SeNPs for 24 h after measurements with NexION 2000 (d) and NexION 300X (e). Dwell time: 100 µs.

**Figure S5.** Data obtained after scICP-MS analyses of ^78^Se in RPTEC/TERT1: time scans for 1:50 dilutions of control cells (a) and cells incubated with cisplatin and Ch-SeNPs for 24 h (b), including an amplified single cell pulse for the second case (c); and histograms of mass distribution (d, e) for cells co-treated with cisplatin and Ch-SeNPs for 24 h after measurements with NexION 2000 (d) and NexION 300X (e). Dwell time: 100 µs.

**Figure S6.** Plots showing the variation of the number of single cell events (a, c) and the average element content per cell (b, d) with the dwell time after scICP-MS analyses of ^195^Pt (a, b) and ^78^Se (c, d) in RPTEC/TERT1 co-treated 24 h with cisplatin and Ch-SeNPs (1:100 dilution). Results are presented as the mean ± the standard deviation (n = 3).

**Figure S7.** Plots showing the variation of the average element content per cell with the number of pellet washes during bulk analyses of ^195^Pt (a) and ^78^Se (b) in RPTEC/TERT1 co-treated 24 h with cisplatin and Ch-SeNPs. Results are presented as the mean ± the standard deviation (n = 3).

**Table S1.** Conditions used for bulk ICP-MS (a) and scICP-MS (b) analysis

**a)**

| **Operating conditions for bulk ICP-MS analysis** | | | |
| --- | --- | --- | --- |
| ***ICP-MS*** | Agilent 7700x | ***Nebulizer*** | Concentric (Meinhard) |
| ***RF power (W)*** | 1500 | ***Dwell time (ms)*** | 100 |
| ***Plasma gas flow rate (L min^-1^)*** | 15 | ***Spray chamber*** | Scott double-pass type |
| ***Nebulizer gas flow rate (L min^-1^)*** | 1 | ***Mode*** | Standard |
| ***Auxiliary gas flow rate (L min^-1^)*** | 1 | ***Isotopes analysed*** | ^195^Pt, ^78^Se, ^193^Ir (ISTD), ^89^Y (ISTD) |

**b)**

| **Operating conditions for scICP-MS analysis** | | |
| --- | --- | --- |
| ***ICP-MS*** | NexION 2000 | NexION 300X |
| ***RF power (W)*** | 1600 | 1600 |
| ***Plasma gas flow rate (L min^-1^)*** | 15 | 15 |
| ***Nebulizer gas flow rate (L min^-1^)*** | 0.35 | 0.9 |
| ***Make up gas flow rate (L min^-1^)*** | 0.70 | 1.2 |
| ***Sample flow rate (µL min^-1^)*** | 10 | 100 |
| ***Transport efficiency (%)*** | > 40 | < 8 |
| ***Dwell time (µs)*** | 50-200 | 50-200 |
| ***Acquisition time (s)*** | 100 | 100 |
| ***Nebulizer*** | CytoNeb with PFA gas line | Concentric (Meinhard) |
| ***Spray chamber*** | Asperon spray chamber | Cyclonic spray chamber |
| ***Mode*** | Standard | Standard |
| ***Isotopes analysed*** | ^195^Pt, ^78^Se | ^195^Pt, ^78^Se |

**Table S2.** Estimated reduction in the accumulation of intracellular Pt after 24 and 48 h cotreatment with cisplatin and Ch-SeNPs, SeMet or Met, taking as reference the uptake rate of cisplatin alone (a). Estimated reduction in the accumulation of intracellular Se after 24 and 48 h co-treatments with cisplatin and Ch-SeNPs or SeMet, taking as reference the uptake rate of each selenocompound alone (b). Calculations are based on the mean values of the intracellular content of Pt and Se obtained for RPTEC/TERT1 and HeLa after scICP-MS and bulk ICP-MS analyses (n = 3).

**a)**

| **Treatment** | **Cell line** | **Incubation time (h)** | **Reduction in Pt accumulation (%)** | | **Difference (%)** |
| --- | --- | --- | --- | --- | --- |
|  |  |  | ***Single cell analysis*** | ***Bulk analysis*** |  |
| **Cisplatin + Ch-SeNPs** | **RPTEC/TERT1** | **24** | < 5 | < 5 | - |
|  |  | **48** | < 5 | < 5 | - |
|  | **HeLa** | **24** | < 5 | < 5 | - |
|  |  | **48** | < 5 | < 5 | - |
| **Cisplatin + SeMet** | **RPTEC/TERT1** | **24** | 35 | 30 | -14 |
|  |  | **48** | 55 | 49 | -11 |
|  | **HeLa** | **24** | 26 | 33 | 27 |
|  |  | **48** | 34 | 41 | 21 |
| **Cisplatin + Met** | **RPTEC/TERT1** | **24** | 31 | 36 | 16 |
|  |  | **48** | 46 | 40 | -13 |
|  | **HeLa** | **24** | 32 | 25 | -22 |
|  |  | **48** | 44 | 37 | -16 |

**b)**

| **Treatment** | **Cell line** | **Incubation time (h)** | **Reduction in Se accumulation (%)** | | **Difference (%)** | |
| --- | --- | --- | --- | --- | --- | --- |
|  |  |  | ***Single cell analysis*** | ***Bulk analysis*** |  |  |
| **Cisplatin + Ch-SeNPs** | **RPTEC/TERT1** | **24** | 20 | 12 | -40 |  |
|  |  | **48** | 54 | 37 | -31 |  |
|  | **HeLa** | **24** | 46 | 62 | 35 |  |
|  |  | **48** | 67 | 83 | 24 |  |
| **Cisplatin + SeMet** | **RPTEC/TERT1** | **24** | 39 | 48 | 23 |  |
|  |  | **48** | 56 | 66 | 18 |  |
|  | **HeLa** | **24** | 34 | 46 | 35 |  |
|  |  | **48** | 61 | 74 | 21 |  |
